# Altered Cerebellar-Cerebral Circuits in Patients With Type 2 Diabetes Mellitus

**DOI:** 10.3389/fnins.2020.571210

**Published:** 2020-09-24

**Authors:** Dongsheng Zhang, Fei Qi, Jie Gao, Xuejiao Yan, Yarong Wang, Min Tang, Xia Zhe, Miao Cheng, Man Wang, Qingming Xie, Yu Su, Xiaoling Zhang

**Affiliations:** ^1^Department of MRI, Shaanxi Provincial People’s Hospital, Xi’an, China; ^2^Department of Graduate, Xi’an Medical University, Xi’an, China; ^3^Department of Diagnostic Radiology, The First Affiliated Hospital of Xi’an Jiaotong University, Xi’an, China

**Keywords:** type 2 diabetes mellitus, resting-state fMRI, functional connectivity, cerebellum, subregion, neuroimaging

## Abstract

The role of the cerebellum in type 2 diabetes mellitus (T2DM) has been receiving increased attention. However, the functional connectivity (FC) between the cerebellar subregions and the cerebral cortex has not been investigated in T2DM. Therefore, the purpose of this study was to investigate cerebellar-cerebral FC and the relationship between FC and clinical/cognitive variables in patients with T2DM. A total of 34 patients with T2DM and 30 healthy controls were recruited for this study to receive a neuropsychological assessment and undergo resting-state FC. We selected four subregions of the cerebellum (bilateral lobules IX, right and left Crus I/II, and left lobule VI) as regions of interest (ROIs) to examine the differences in cerebellar-cerebral circuits in patients with T2DM compared to healthy controls. Correlation analysis was performed to examine the relationship between FC and clinical/cognitive variables in the patients. Compared to healthy controls, patients with T2DM showed significantly decreased cerebellar-cerebral FC in the default-mode network (DMN), executive control network (ECN), and visuospatial network (VSN). In the T2DM group, the FC between the left cerebellar lobule VI and the right precuneus was negatively correlated with the Trail Making Test A (TMT-A) score (*r* = −0.430, *P* = 0.013), after a Bonferroni correction. In conclusion, patients with T2DM have altered FC between the cerebellar subregions and the cerebral networks involved in cognitive and emotional processing. This suggests that a range of cerebellar-cerebral circuits may be involved in the neuropathology of T2DM cognitive dysfunction.

## Introduction

Type 2 diabetes mellitus (T2DM) is a risk factor for Alzheimer’s disease (AD) and vascular dementia (Curb et al., 1999; Luchsinger et al., 2001; Cukierman et al., 2005). It causes emotional abnormalities and multiple cognitive dysfunctions, such as executive function and visual space (Macpherson et al., 2017). Brain network disorders and abnormal neuronal activity are the neural bases of cognitive impairment. Studies have found, for example, that disruption of the default-mode network (DMN) may be related to episodic memory impairment (Qi et al., 2017) and depression (Cui et al., 2015), whereas disruption of the executive control network (ECN) may lead to reduced working memory (Alvarenga et al., 2010) in patients with T2DM. In addition, T2DM studies have demonstrated abnormal neuronal activity in the core regions (posterior parietal and occipital cortex) of the visuospatial network (VSN) (Cui et al., 2014; Peng et al., 2016; Wang et al., 2017).

Many neuroimaging studies on the cognitive networks have mainly focused on the cerebrum. Several clinical and neuroimaging studies have demonstrated that the cerebellum is also involved in multiple cognitive functions, such as working memory, executive function, emotional processing, and attention (Middleton and Strick, 2000; Timmann and Daum, 2007; Baillieux et al., 2010; Grimaldi and Manto, 2012). Moreover, different cerebellar subregions have distinct functional heterogeneity, and some cerebellar subregions have functional connectivity (FC) with networks related to cognition. Habas et al. (2009) found that the right and left cerebellar Crus I/II interact with cerebral executive control circuitry contralaterally, and bilateral lobule IX with the DMN, which was confirmed by later studies (Sang et al., 2012; Li et al., 2013; Marien and Beaton, 2014). In addition, a meta-analysis of neuroimaging studies showed the left lobule VI is engaged in visuospatial processing (Stoodley and Schmahmann, 2009). Furthermore, patients with cerebellar lesions have been found to experience abnormal executive functions (Karatekin et al., 2000), visuospatial dysfunction (Levisohn et al., 2000), and depressive symptoms (Kim et al., 2017).

Although accumulating evidence has highlighted the involvement of the cerebellum in cognitive function, studies exploring the potential role of the cerebellum in T2DM are relatively rare. A plausible reason is that the cerebellum is thought to be protected by hypoglycemia (Ertas et al., 1997) and may prevent damage from hyperglycemia (Heikkila et al., 2010), although such theories are being challenged by some recent studies. For example, it has been found that the density of cerebellar gray matter (Garcia-Casares et al., 2014) and blood flow (Dai et al., 2017) are decreased in patients with T2DM. In addition, several studies that used resting-state functional magnetic resonance imaging (fMRI) have demonstrated abnormalities in the spontaneous activity of neurons in the cerebellum, and impairment of FC among other regions in patients with T2DM (Xia et al., 2013; Cui et al., 2014; Garcia-Casares et al., 2014; Peng et al., 2016; Tan et al., 2019). These studies provide evidence that the cerebellum may be a vulnerable region in T2DM.

It is currently accepted that the core neuropathological features in the AD brain are extensive extracellular neuritic amyloid plaques leading to dystrophic neurites and intracellular neurofibrillary tangles consisting of tau proteins; these hallmarks ultimately lead to synapse atrophy and neuron loss (Lemche, 2018). The pathological mechanism of cognitive impairment in T2DM is believed to be similar to that of AD (Bedse et al., 2015). That is, insulin resistance leads to the phosphorylation of tau and the production of amyloid-β plaques, which tend to accumulate in cognitively related cortical hubs, disrupting the connectivity among these regions, and thus, causing cognitive impairment (Buckner et al., 2009). Therefore, T2DM-induced cognitive decline may be due to a disconnection syndrome. Resting-state FC can be used to evaluate interregional cooperation between different brain regions. Previous studies that examined different regions of interest (ROIs) other than the cerebellum have revealed the abnormal patterns of FC in T2DM (Musen et al., 2012; Chen et al., 2015; Sun et al., 2018).

The cerebellum appears to suffer from neuropathological damage, as the cerebrum does, because amyloid-β plaque deposits have been found in the cerebellum of patients with AD (Ciavardelli et al., 2010). Furthermore, primate research has found that phosphorylated tau is increased in the cerebellum of diabetic monkeys (Morales-Corraliza et al., 2016). However, it is still unclear whether the cerebellum, together with the cerebrum, participates in cognitive impairment in T2DM. Fang et al. (2017) found that diabetes-related cognitive deficits are associated with abnormal cerebellar-cerebral circuits, structurally. To the best of our knowledge, however, no study has investigated functional changes in cerebellar-cerebral circuits in patients with T2DM. Therefore, it is crucial to explore the intrinsic FC patterns of the cerebellum in T2DM.

The purpose of the present study was to investigate the intrinsic FC of cerebellar subregions related to cognitive function in patients with T2DM using resting-state fMRI, and reveal the neuropathological mechanisms of cerebellar-related cognitive impairment in T2DM. We hypothesized that patients with T2DM have decreased cerebellar-cerebral FC that is associated with cognitive impairment.

## Materials and Methods

### Participants

Thirty-eight patients with T2DM from the Department of Endocrinology of Shaanxi Provincial People’s Hospital and 33 euglycemic healthy controls from the local community were enrolled in our study from March 2018 to May 2019. All the participants were 40–70 years old, right-handed, and received at least 6 years of education. The patients met the diagnostic criteria of T2DM according to the American Diabetes Association in 2014, and were on stable therapy (diet, oral medications, and/or insulin). Patients were excluded from the study if they had a history of hypoglycemic episodes and macrovascular diseases (e.g., cerebrovascular or cardiovascular diseases), or had hypoglycemia (blood glucose <3.9 mmol/L) or hyperglycemia (blood glucose >33.3 mmol/L) during their hospital stay. The exclusion criteria of both groups were a self-reported history of known brain injury, epilepsy, stroke, alcohol or other substance dependence, Parkinson’s disease, major depression or other disorders that could affect cognitive function, major medical illnesses (e.g., cancer), or MRI contraindications. Details of the complications and therapeutic agents for T2DM are provided in Supplementary Tables S1, S2.

All patients were instructed to control their blood glucose according to their doctor’s orders on the day of the scan. They arrived at the Department of MRI between 6:30 and 7: 00 pm after dinner. A structured clinical interview and a series of psychological tests were conducted for approximately 30 min. Then, the patients were prepared for the MRI scan. Only one patient was scheduled each day to ensure that each patient’s MRI scan time was completed between 7:30 and 8:30 pm. Healthy controls had to have normal fasting glucose levels, and their test procedure and scan time were consistent with those of the patients. All the participants were awake during the scan and did not feel discomfort. The study was approved by the Ethics Committee of Shaanxi Provincial People’s Hospital. The study protocol was explained to all the participants and signed informed consent was obtained before they participated in the study.

### Clinical Data and Neuropsychological Tests

The medical history and clinical data of the patients were obtained from the medical records and questionnaires. The clinical data of the healthy controls were collected from the outpatient medical examination center, and included weight, height, blood pressure, and body mass index. Blood pressure was measured while sitting at three different times during the day and then averaged. Blood samples were obtained, after overnight fasting for at least 8 h, to test the levels of fasting blood glucose, triglycerides, total cholesterol, low-density lipoprotein, and glycated hemoglobin. A battery of neuropsychological tests was used to evaluate the participants’ mental status and cognitive domains. The Mini Mental State Examination and the Montreal Cognitive Assessment were used to assess general cognitive function. Information processing speed and attention were tested by the Trail Making Test A (TMT-A). Executive function and visuospatial skills were evaluated by the Clock Drawing Test, and the Beck Depression Inventory was used to evaluate depressive symptoms. The neuropsychological tests were performed by a psychiatrist with more than 5 years of work experience.

### Image Acquisition

The MRI scan was performed on a 3.0-Tesla MR scanner (Philips Ingenia, Best, Netherlands) using a 16-channel phased-array head coil. All participants were instructed to keep their eyes closed but stay awake during the scan. Foam pads and headphones were used to control head motion and decrease scanner noise as much as possible. Conventional T2WI and FLAIR scans were also acquired to exclude visible brain lesions. Sagittal 3-dimensional T1-weighted images were acquired with the following parameters: TR = 7.5 ms, TE = 3.5 ms, FA = 8°, FOV = 250 mm × 250 mm, matrix = 256 × 256, slice thickness = 1 mm, no gap, and 328 sagittal slices. Resting-state functional BOLD images were obtained using a gradient-echo planar sequence with the following parameters: TR = 2000 ms, TE = 30 ms, slices = 34, thickness = 4 mm, gap = 0 mm, FOV = 230 mm × 230 mm, matrix = 128 × 128, FA = 90°, and 200 volumes.

### Imaging Data Analysis

Functional data analyses were conducted using the programs in Data Processing & Analysis for Brain Imaging v3.0^1^ that are based on Statistical Parametric Mapping v12 (SPM12).^2^ The slice timing and realignment for head motion correction were performed after discarding the first 10 time points. Any head motion >1.5 mm or translation >1.5° rotation in any direction was excluded. Then, normalization was performed based on the resulting images using a unified segmentation of anatomical images (resampling voxel size = 3 mm × 3 mm × 3 mm). Multiple regression models were used to remove the effects of covariates of no interest, which involved 24 motion parameters, cerebrospinal fluid signals, and white matter signals. The obtained images were smoothened using an isotropic Gaussian smooth kernel with a FWHM of 6, followed by detrending and filtering (0.01–0.08 Hz).

**FIGURE 1:**
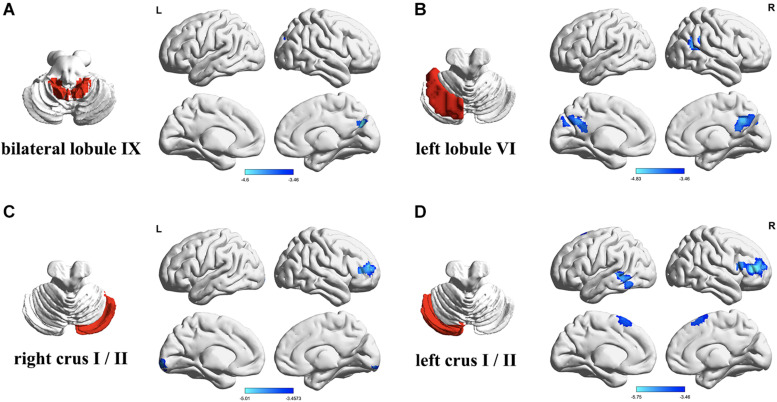
Differences in resting-state functional connectivity between the patients with type 2 diabetes mellitus (T2DM) and the healthy controls (two-sample *t*-test, *P* < 0.05, Gaussian random field-corrected). **(A)** Lower connectivity of bilateral lobule IX in T2DM. **(B)** Lower connectivity of left lobule VI in T2DM. **(C)** Lower connectivity of right Crus I/II in T2DM. **(D)** Lower connectivity of left Crus I/II in T2DM. L, left; R, right.

Four ROIs were defined in standard MNI space using the map of the Automated Anatomical Labeling atlas. The ROIs were the bilateral cerebellar lobules IX, the left and right cerebellar Crus I/II, and the left cerebellar lobule VI (Figure 1). Correlation analysis was performed between the mean signal change and the time series of every voxel of the whole brain for each ROI. The resulting correlation coefficients (*r*) were converted by the Fisher’s *r*-to-*z* transformation to improve the Gaussianity of their distribution. The ROIs and intergroup analysis results were visualized using the BrainNet Viewer package.^3^

An age-related white matter change scale was used to quantitatively evaluate lacunar infarcts and white matter hyperintensity based on fluid attenuated inversion recovery images, excluding subjects with a rating score >2. Seven participants were excluded from the final statistical analysis: four (three with T2DM and two controls) were excluded for excessive motion, and two (one with T2DM and one control) were excluded for a rating score of white matter hyperintensity >2.

### Statistical Analysis

Statistical analyses were performed using SPSS v17.0 (SPSS Inc., Chicago, IL, United States). Independent sample *t*-tests (two-tailed) were used to determine group differences for normally distributed variables. Variables that were not normally distributed were analyzed using the Mann–Whitney *U*-test. The chi-square (χ^2^) test was used to assess intergroup differences in gender and smoking. The significance level was set at *P* < 0.05.

Voxel-wise two-sample *t*-tests embedded in Data Processing & Analysis for Brain Imaging were performed to evaluate group differences in resting-state FC for each ROI after controlling for years of education. The significance was determined using Gaussian random field correction with *P* < 0.05 (voxel *P* < 0.001, cluster size >46).

The mean FC values of the functionally altered brain areas between the groups were extracted from the patients with T2DM. Partial correlation analyses were conducted to identify the relationships between the mean FC and clinical/cognitive variables, by controlling for years of education.

## Results

### Clinical and Neuropsychological Data

A total of 34 patients with T2DM and 30 healthy controls were enrolled in the study. The demographic, clinical, and cognitive data of the two groups are presented in Table 1. There were no significant group differences in age, gender, smoking, body mass index, total cholesterol, triglycerides, low-density lipoprotein, blood pressure, or cognitive scores (*P* > 0.05). However, level of education was higher in the control group than the T2DM group (*P* < 0.01). In addition, the T2DM group had increased levels of fasting blood glucose, glycated hemoglobin, and Beck Depression Inventory score compared to the control group (all *P-*values < 0.001). In the T2DM group, there were 15 patients with no complications and 19 patients with complications including nephropathy, peripheral neuropathy, retinopathy (Supplementary Table S1). Eleven patients received dietary restriction, two received insulin, 16 patients received oral medication (including metformin, sulfonylureas, and acarbose), and five patients received a combination of insulin and oral medication (Supplementary Table S2). Insulin was administered via subcutaneous injection.

**TABLE 1:** Demographic, clinical, and cognitive data of the patients with type 2 diabetes mellitus (T2DM) and the healthy controls.

Variable	T2DM (*n* = 34)	Controls (*n* = 30)	*P*-value
Age (years)	56.18 ± 5.74	54.41 ± 5.31	0.21
Male/female	25/9	20/10	0.55^#^
Educational level (years)	12.88 ± 2.92	15.14 ± 2.34	< 0.01*
Diabetes duration (years)	11.12 ± 6.02	–	–
Systolic BP (mmHg)	130.03 ± 17.92	123.45 ± 9.36	0.08
Diastolic BP (mmHg)	81.79 ± 10.23	82.41 ± 6.50	0.78
BMI (kg/m^2^)	25.25 ± 2.44	24.74 ± 2.94	0.45
FBG (mmol/L)	9.49 ± 3.14	5.55 ± 0.81	< 0.01*
HbA1c (%)	8.09 ± 1.84	5.62 ± 0.54	< 0.01*
TG (mmol/L)	1.51 ± 0.62	1.89 ± 1.40	0.16
TC (mmol/L)	4.68 ± 1.55	4.93 ± 0.92	0.44
LDL (mmol/L)	2.44 ± 0.73	2.84 ± 0.86	0.06
Total/smoking	34/21	30/16	0.50^#^
TMT-A	70.38 ± 28.70	64.17 ± 19.83	0.33
MMSE	27.82 ± 2.45	28.10 ± 1.82	0.61
MoCA	26.18 ± 2.44	27.00 ± 1.73	0.16
CDT	16.64 ± 7.94	19.45 ± 6.01	0.13
BDI	0 (0.12)	0 (0.5)	0.04⁢*Δ

*Normally distributed variables are presented as mean ± standard deviation and variables that are not normally distributed are presented as median (minimum, maximum). BMI, body mass index; FBG, fasting blood glucose; TG, triglycerides; TC, total cholesterol; LDL, low-density lipoprotein; HbA1c, glycated hemoglobin; MMSE, Mini Mental State Examination; MoCA, Montreal Cognitive Assessment; TMT-A, Trail Making Test A; CDT, Clock Drawing Test; BDI, Beck Depression Inventory. ^Δ^*P* for the Mann–Whitney *U*-test; ^#^*P* for the χ^2^ test; ^∗^*P* < 0.05.*

### The FC Between Cerebellar Subregions and the Cerebral Cortex

The FC between the bilateral cerebellar lobules IX and the right cuneus/precuneus was significantly lower in the T2DM group than the control group, after controlling for years of education (*P* < 0.05). The T2DM group also had significantly lower FC (all *P-*values < 0.05): (a) between the right cerebellar Crus I/II and the right dorsolateral prefrontal cortex and right lingual; (b) between the left cerebellar Crus I/II and the right dorsolateral prefrontal cortex, left middle/inferior temporal gyrus, and bilateral supplementary motor area. In addition, the T2DM group had significantly lower FC between the left cerebellar lobule VI and the bilateral precuneus/posterior cingulate, right angular gyrus/supramarginal gyrus, and right superior/middle temporal gyrus. We did not find any regions in which FC was significantly higher in the T2DM group than the control group (Table 2 and Figure 1).

**TABLE 2:** Abnormal functional connectivity in the patients with type 2 diabetes mellitus group compared to the healthy controls.

Seed ROI	Brain region	Peak MNI coordinates			Voxel (mm^3^)	BA	*t*-value
		X	Y	Z			
B cerebellar lobules IX	R cuneus/precuneus	18	−72	30	54	7	−4.57
R cerebellar Crus I/II	R dlPFC	36	54	15	114	10/46	−4.98
	R lingual	6	−93	−15	58	18	−4.65
L cerebellar Crus I/II	R dlPFC	51	42	12	182	10/46	−5.75
	L middle/inferior temporal gyrus	−57	−42	0	85	20/21	−4.99
	B supplementary motor area	3	18	69	52	6	−4.35
L cerebellar lobule VI	B precuneus/posterior cingulate	6	−54	33	129	7	−4.49
	R angular gyrus/supramarginal gyrus superior/middle temporal gyrus	51	−48	30	84	30/40/13	−4.53

*BA, Brodmann’s area; MNI, Montreal Neurological Institute; dlPFC, dorsolateral prefrontal cortex; B, bilateral; L, left; R, right. Group differences in functional connectivity were evaluated by two-sample *t*-test after controlling for years of education (*P* < 0.05, Gaussian random field-corrected).*

### Correlation Between the FC and Clinical/Cognitive Variables

**FIGURE 2:**
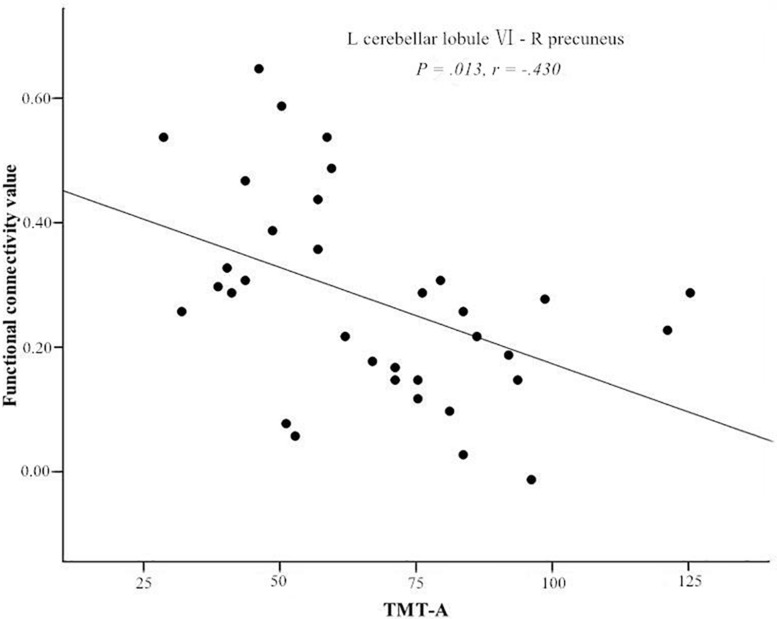
Significant negative correlations of the Trail Making Test A (TMT-A) score and the functional connectivity between left cerebellar lobule VI and right precuneus. L, left; R, right.

After controlling for years of education (*P* < 0.05). The FC between the left cerebellar lobule VI and the right precuneus was negatively correlated with TMT-A score (*r* = −0.430, *P* = 0.013), after the Bonferroni correction for *P* (Figure 2). There were no significant correlations between the cerebellar-cerebral FC and other clinical/cognitive variables.

## Discussion

This study explored the patterns of resting-state cerebellar-cerebral FC in T2DM. We found that cerebellar-cerebral FC decreased in the patients with T2DM compared to healthy controls, including the DMN, ECN, and VSN. This result suggests that cognitive-related cerebellar subregions are involved in the neuropathology of cognitive impairment in T2DM.

### Decreased Connectivity of the Cerebellum-Cerebral DMN in T2DM

The DMN is involved in cognitive functions such as episodic memory retrieval, self-referencing, and emotion management (Buckner et al., 2008). Previous studies have found decreased gray-matter volume in DMN-related regions (Liu et al., 2017), and abnormal FC within the DMN as well as between the DMN and other brain regions (including the cerebellum) in patients with T2DM (Zhou et al., 2010; Musen et al., 2012; Zhang et al., 2015; Yang et al., 2016). The present study provides further evidence for the impairment of the cerebellum-cerebral DMN connection in patients with T2DM, especially a disconnection between the bilateral cerebellar region IX and the right cuneus/precuneus.

The precuneus is a pivotal hub of the DMN and plays a vital role in emotion management (Sheline et al., 2009). The cerebellum is also involved in emotion regulation, and abnormal connectivity between the left cerebellum and the posterior cingulate cortex/precuneus might be a predictor of suicidal behavior in depressed adolescent patients (Zhang et al., 2016). Several studies suggest that the relationship between depression and T2DM is bi-directional or co-morbid (Mezuk et al., 2008; O’Connor et al., 2009; Stuart and Baune, 2012). The depression scores of our patients with T2DM were significantly higher than that of healthy participants, which suggests that the patients may be prone to depression. Therefore, the decreased FC between the cerebellar region IX and the precuneus might underlie the dysregulation of emotion.

### Decreased Connectivity of the Cerebellum-Cerebral ECN in T2DM

Multiple studies have found that biphasic projections and functional coupling exist in the prefrontal cortex and cerebellar Crus I/II (Middleton and Strick, 1994; Kelly and Strick, 2003; Strick et al., 2009). Salmi et al. (2010) showed that the cerebellar Crus I/II is linked to lateral prefrontal cortex activity with increased working memory task load. In addition, the supplementary motor area is considered to be involved in the executive system of attention and responsible for the planning of motor actions (Marek et al., 2010). Here, we demonstrated that the cerebellar Crus I/II had decreased FC with the dorsolateral prefrontal cortex and supplementary motor area. This result suggests that the bilateral cerebellum-cerebral ECN circuit is disconnected in T2DM.

Interestingly, we found that the right cerebellar Crus I/II had decreased FC with the ipsilateral rather than the contralateral dorsolateral prefrontal cortex. This result is contradictory to the theory that the bilateral Crus I/II is coupled with the contralateral ECN. Krienen and Buckner (2009) found that the cerebellar Crus I/II was slightly connected (20–30%) to the ipsilateral prefrontal cortex in healthy individuals. Therefore, we speculate that the abnormal FC in T2DM may be related to damage to the inherent pattern of the ECN. Structural research has also found decreased connectivity between the right Crus II and the right superior frontal gyrus instead of the left (Fang et al., 2017). Based on the consistent results of structural and functional studies, we believe that altered cerebellum-cerebral ECN is mainly manifested in cross-projecting functional abnormalities in T2DM.

Several studies have found that the lingual gyrus and lateral temporal lobes participate in and maintain working memory processing (Zamora et al., 2016; Moon and Jeong, 2017; Fan et al., 2019). A study on a similar diabetic population also reported an association between poor working memory in patients and disrupted connectivity of the occipital network anchored in the lingual gyrus (Cui et al., 2016). Therefore, the lower FC between the cerebellar Crus I/II and the lingual gyrus and lateral temporal gyrus may indicate impaired working memory in T2DM.

### Decreased Connectivity of the Cerebellum-Cerebral VSN in T2DM

Cerebellar lobule VI receives peripheral and central inputs from the visual systems (Schmahmann, 2019). Based on independent component analysis, previous studies have shown that the posterior parietal cortex (angular gyrus and supramarginal gyrus), precuneus, and posterior cingulate are the main regions of the VSN (Beckmann et al., 2005; Smitha et al., 2017). Many studies reported that the VSN has abnormal neural intensity, reduced gray matter volume, and/or decreased perfusion of the precuneus and posterior cingulate (Cui et al., 2014; Peng et al., 2016; Liu et al., 2017; Wang et al., 2017; Wu et al., 2017), which is associated with impaired visuospatial function in T2DM (Cui et al., 2017). Here, our results suggest that the left cerebellar lobule VI is related to the impairment of visuospatial function in patients with T2DM.

Trail Making Test A is not only useful for assessing neural flexibility and attention, but it may be a particularly useful tool for detecting changes in visuospatial abilities (Vaportzis et al., 2019). In the T2DM group, we found that the decreased FC between the left cerebellar lobule VI and the right precuneus was negatively correlated with TMT-A score. This result is consistent with a tractography study of T2DM (Fang et al., 2017), which also found decreased anatomical connections between cerebellar lobule VI and the precuneus. The bilateral lobule VI and the precuneus participate in memory-guided visuospatial attention processes and have considerably greater activity during such processing (Rosen et al., 2018). The left cerebellar lobule VI may, in fact, play a vital role in controlling visuospatial attention (Striemer et al., 2015). Therefore, the abnormal connection between the left cerebellar lobule VI and the precuneus is indicative of impaired visuospatial attention in patients with T2DM.

In normal elderly individuals, amyloid-β deposition is most likely to occur in the core regions of DMN such as the precuneus (Mormino et al., 2011). In T2DM patients, insulin resistance can further accelerate amyloid-β and tau deposition (Verdile et al., 2015), increasing the possibility of T2DM developing into AD. Studies have found that the typical AD tau pattern mainly involves the dorsal attention and higher visual network (Hansson et al., 2017). Considering the similar pathological mechanism of T2DM and AD, we speculated that this may be the neural basis of abnormal visuospatial attention in patients with T2DM. Whether the FC disruption of these brain regions is related to abnormal amyloid-β and tau deposition in T2DM will be investigated in our future research.

### Limitations

Several limitations of this study should be mentioned. First, there were no significant group differences in cognitive scores, which may be related to the small sample size of the study. Second, the educational level of the healthy controls was significantly higher than that of the T2DM group. This difference may produce a certain bias in the results, although the educational level is not the only factor that affects cognitive function (Crowe et al., 2013), and we have controlled it in the data analysis. Third, many of the patients received various medications with or without insulin, and therefore, we could not control the effects of medication and insulin on the results. For instance, metformin may affect the cognitive functions of patients with diabetes (Moore et al., 2013). In addition, seven patients included in this study were treated with subcutaneous insulin injection to better control blood glucose, although peripheral insulin may be involved in the modulation of plasma and cerebrospinal fluid amyloid-β levels and cognitive function, theoretically (Morris and Burns, 2012). However, more studies suggest that peripheral insulin therapy is often related to muscle and fat metabolism (Russell-Jones and Khan, 2007; McFarlane, 2009). Fourth, the serum glucose level of patients was not measured immediately before the MRI scan, and future studies should examine the relationship between blood glucose control and neuronal dysfunction. Finally, most of the patients with T2DM in this study were re-visiting the hospital and had a relatively long duration of disease; hence, the results may not be applicable to patients with early T2DM.

## Conclusion

This study identified patterns of abnormal FC between cognitive-related cerebellar subregions and the cerebrum in patients with T2DM. The results suggest that a range of cerebellar-cerebral circuits may be involved in the neuropathology of T2DM, which indicates new directions for exploring cognitive dysfunction in T2DM. Further clinical studies are needed to confirm whether treatments that target the cerebellar-cerebral circuits can improve the cognitive functioning of patients with T2DM.

## Data Availability Statement

The raw data supporting the conclusions of this article will be made available by the authors, without undue reservation.

## Ethics Statement

The studies involving human participants were reviewed and approved by the Ethics Committee of Shaanxi Provincial People’s Hospital. The patients/participants provided their written informed consent to participate in this study.

## Author Contributions

DZ and FQ drafted the manuscript, designed the experiment, and performed the statistical analysis. JG contributed to performing the experiments and revised the manuscript. MT, XZ, and XY collected the data. MC, MW, QX, and YS provided technical support. YW contributed to the manuscript review and critique. XZ made contributions to the design of the experiment and revised the manuscript. All authors read and approved the final manuscript.

## Conflict of Interest

The authors declare that the research was conducted in the absence of any commercial or financial relationships that could be construed as a potential conflict of interest.

## Footnotes

1 http://rfmri.org/dpabi

2 http://www.fil.ion.ucl.ac.uk/spm

3 http://www.nitrc.org/projects/bnv/

## Abbreviations

AD: Alzheimer’s disease
DMN: default-mode network
ECN: executive control network
FC: functional connectivity
ROI: region of interest
T2DM: type 2 diabetes mellitus
TMT-A: Trail Making Test A
VSN: visuospatial network.
