# Supplementary material for: Altered Cerebellar-Cerebral Circuits in Patients With Type 2 Diabetes Mellitus
Source: Front Neurosci. 2020 Sep 24;14:571210. doi: 10.3389/fnins.2020.571210 (PMC7541847; doi:10.3389/fnins.2020.571210)
Supplement: Supplementary file 1 [file Table_1.pdf]

TABLE S1: Complications of type 2 diabetes mellitus in the patients included in this study

Complication	Number of patients
No complications	15
Nephropathy	7
Peripheral neuropathy	4
Retinopathy	5
Nephropathy + peripheral neuropathy	3

TABLE S2: Therapeutic agents for type 2 diabetes mellitus in the patients included in this study

Therapeutic agent	Medication	Number of patients
Dietary restriction		11
Insulin		2
Oral medication	Metformin	9
	Metformin + sulfonylureas	3
	Metformin + acarbose	3
	Sulfonylureas	1
Insulin + oral medication	Metformin	3
	Acarbose	1
	Metformin + sulfonylureas	1
